# Metabolite and Elastase Activity Changes in Beach Rose (*Rosa rugosa*) Fruit and Seeds at Various Stages of Ripeness

**DOI:** 10.3390/plants10071283

**Published:** 2021-06-24

**Authors:** Seung-Hun Chae, Young-Sang Lee, Jin-Hee Kim, Tae-Ho Han, Kang-Mo Ku

**Affiliations:** 1 BK21 Interdisciplinary Program in IT-Bio Convergence System, Chonnam National University, Gwangju 61186, Korea; kjcmc0921@naver.com (S.-H.C.); hth@jnu.ac.kr (T.-H.H.); 2 Department of Horticulture, Chonnam National University, Gwangju 61186, Korea; 3 Department of Medical Biotechnology, Soonchunhyang University, Asan 31538, Korea; Mariolee@sch.ac.kr; 4 Department of Herbal Skin Care & Cosmetology, Daegu Haany University, Gyeongsan 38610, Korea; gonogo1@nate.com; 5 Healinnols, Inc., Daejeon 34054, Korea

**Keywords:** antioxidant, elastase inhibitory activity, fatty acid, ripening stages, *Rosa rugosa*, rose hip, primary metabolite

## Abstract

Rose hips are the fruits of the beach rose (*Rosa rugosa).* To determine the optimal harvest time and to obtain the maximum functional compounds, rose hips at various stages of ripeness (immature, early, mid, and late) were harvested, and the flesh tissue and seeds were separated. The rose hip flesh showed the highest total phenolic content at the mid-ripeness stage (8.45 ± 0.62 mg/g gallic acid equivalent concentration (dry weight)). The early-, mid-, and late-ripeness stages of rose hip flesh did not show significantly different 2,2-diphenyl-1-picrylhydrazyl antioxidant capacities. The elastase inhibitory activity of the 95% ethanol extract from the rose hip seeds was highest at the mid-ripeness stage; however, the elastase inhibitory activity of the rose hip tissue was not significantly different from that of the seeds. Pathway analysis using MetaboAnalyst showed that sucrose, fructose, and glucose gradually increased as the fruit ripened. Ursolic acid was detected in the seeds but not in the flesh. Of the fatty acids, linoleic acid concentrations were highest in rose hip seeds, followed by linolenic acid, oleic acid, and palmitic acid. Fatty acids and ursolic acid might be the active compounds responsible for elastase inhibitory activity and can be utilized as a functional cosmetic material.

## 1. Introduction

The beach rose (*Rosa rugosa* Thunb.), from which rose hip fruit is harvested, is grown on beaches and slopes in Europe, Asia, and North America [1,2]. Rose hips are used in herbal teas, jams, jellies, and medicines and have traditionally been used to treat inflammation, stomach aches, menoxenia, diarrhea, pain, diabetes mellitus, and hemostasis and are used during labor [3,4]. Rose hips have also been used for colitis, hemoptysis, strokes, paralysis, and dysmenorrhea. Furthermore, rose hip fruits and seeds are used as traditional medicines to prevent infections and to treat colds, influenza-like infections, infectious diseases, and fever [5]. Recently, rose hips have been studied for beauty treatments, as they are rich in vitamin C [6].

The rose hip fruit of the beach rose has been studied for its antioxidant capacity and carotenoid, polyphenol, flavonoid, vitamin, terpenoid, steroid, and tocopherol properties [4,7]. Rose hips are rich in vitamins and minerals (e.g., K and P) [8]. Rose hip powder has an anti-inflammatory effect in osteoarthritis [9,10]. Rose hip powder from seeds and flesh affects cell senescence, skin wrinkling, and aging [11]. Rose hip powder is an important and highly effective raw material that prevents aging and promotes cell longevity [12]. Rose hip seed contains 92% unsaturated fatty acids (oleic acid and linoleic acid) [13]—for which it is often used in beauty treatments—and is a potential industrial food source of vitamin E (tocopherol) [14].

Elastin is a protein that connects tissue in the skin, lungs, and arteries and sustains tissue configuration after stretching or recoiling [15]. Ultraviolet exposure and natural aging trigger the expression of elastases, a type of protease that hydrolyzes the elastin fiber network [16]. For example, neutrophil elastase activity is increased in mouse skin exposed to UV radiation [17]. Several studies have suggested that skin aging and wrinkle formation are related to the degradation of elastin [18]. Therefore, elastase inhibition is an important factor to be considered in the production of cosmetic products. 

The importance of raw materials for the cosmetic industry has been raised by the Nagoya Protocol [3]. The global skin care market is anticipated to be worth USD 189.3 billion by 2025 [19]. Raw materials of high-value products, such as those related to medicine, beauty treatments, and health products, are considered highly important. Thus, this study focused on how the maturity of rose hips separately influences antioxidants, secondary metabolites, fatty acid levels, and elastase inhibitory activity in flesh and seeds. The objective of this research was to identify metabolic materials according to different maturities of rose hips at harvest and to select the appropriate stage of maturity for harvest and use in the cosmetic industry.

## 2. Results and Discussion

### 2.1. Characteristics of Each Growth Stage

**Figure 1:**
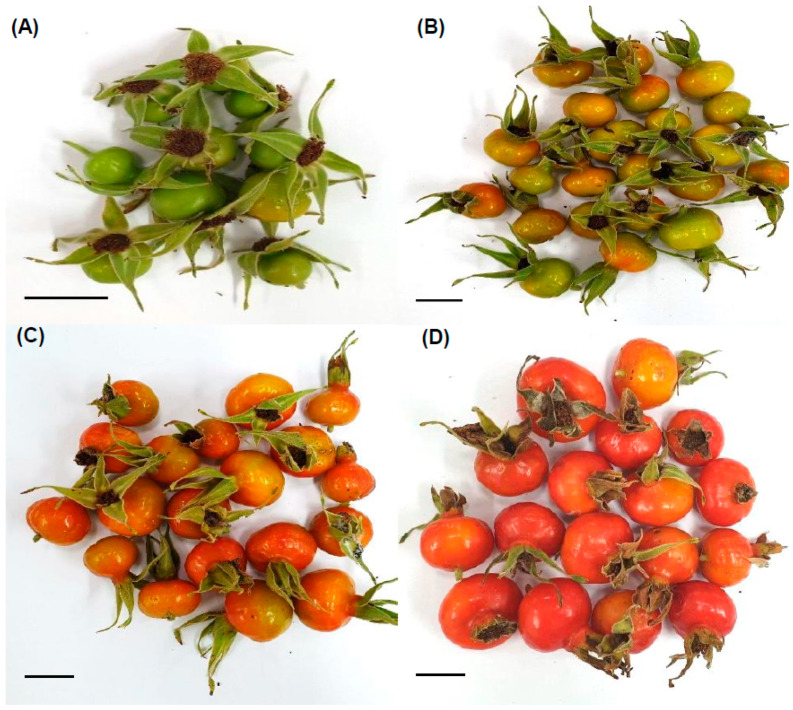
Representative images of rose hips at various ripeness stages. (**A**–**D**) indicate representative images of harvested rose hips from immature and early-, mid-, and late-ripeness stages, respectively. Each scale bar in the lower left of the picture indicates 1 cm.

**Figure 2:**
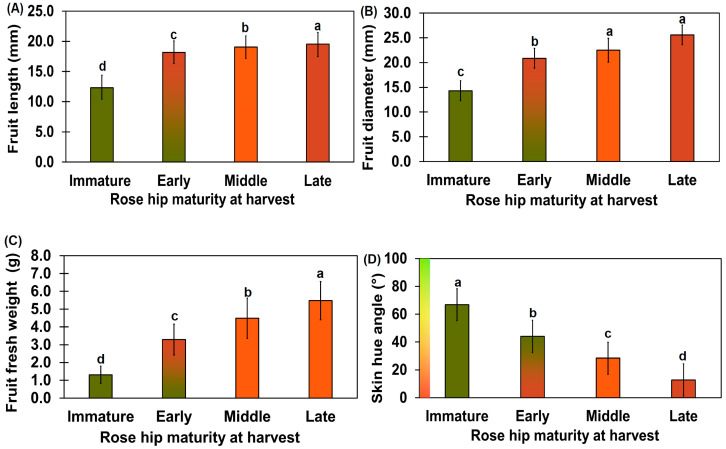
Diameter (**A**), length (**B**), weight (**C**), and surface hue angle (**D**) of rose hips at various ripening stages. The diameter of the rose hip flesh was measured using digimatic calipers at the widest cross-section point (**A**). The length of the rose hip flesh was measured using digimatic calipers at the widest part of the longitudinal section (**B**). The weight of the rose hip flesh was measured using an electronic scale (**C**). The hue angle was determined by converting the RGB values using the OI Color Picker program (https://shaeod.tistory.com/565, accessed on 11 February 2021) and SAL 1650 camera photos (**D**). Different letters above the error bars indicate significant differences according to Tukey’s HSD test (*p* < 0.05). Number of immature rose hip samples = 14; number of early-, mid-, and late-stage rose hip samples = 16.

The *R. rugosa* fruit was classified into four different ripening stages based on the fruit skin color, calyx color, sepal freshness, diameter, length, and weight (Figure 1 and Figure 2). The skin color of the immature fruit samples of *R. rugosa* was mostly green, whereas samples of the other ripening stages were orange (early-ripeness stage) or red (mid- and late-ripeness stages) colors. The mid- and late-ripeness samples had different sepal freshness or dryness; the sepals of the late-ripeness samples were completely dry and brown in color. However, the sepals of the immature and early-ripeness samples were green.

The diameter of the *R. rugosa* fruit at the various ripening stages was significantly different, as per Tukey’s test (*p* < 0.01). The diameter and length of the immature rose hip fruit were 14.3 ± 2.0 and 12.3 ± 1.9 mm, respectively (Figure 2A,B). The diameter and length of the early-ripeness rose hip fruit were 20.9 ± 2.0 and 18.2 ± 1.9 mm, respectively, and were different from those of the immature stage (*p* < 0.05). The fruit length of the mid-ripeness rose hips was not significantly different from those of the late-ripeness rose hips. The fruit diameter and length of rose hips weighing more than 3 g were 17.99 ± 1.44 and 28.98 ± 2.60 mm, respectively [20]. Unlike our rose hips, the rose hips in the afore-mentioned study had an oval shape with a longer diameter, indicating genetic differences. The weight increment rate from the immature stage to the late-ripeness stage decreased slowly (Figure 2C). The weight of the rose hip in this study was twice that of other rose hip genotypes [20].

The immature rose hip fruit surface had a 66.82 ± 3.38° (green) hue angle, whereas the late-ripeness fruit surface had a 12.74 ± 6.13° (red) hue angle (Figure 2D). A previous study reported that the hue angle after 14 d of harvest was 15.39 ± 0.59° [21]. This was close to the late rose hip fruit hue angle reported in our study. The hue angles of all ripening stages were significantly different, as per Tukey’s test (*p* < 0.01).

Uggla et al. [22] reported that yellow and red colors are associated with carotenoid accumulation. Total free carotenoid content of *R. rugosa* fruit was 324.9 ± 30.0 μg/g; mostly β-carotene, lycopene, and some carotenoids were present in esterified forms [23]. Based on a previous study, the hue angle of *R. rugosa* fruit decreased during ripening due to the accumulation of carotenoids, including β-carotene and lycopene [22,23]. The length and weight of the rose hips (*Rosa rugosa* Thumb.) in this study indicate suitability for a cosmetic industry resource.

### 2.2. Total Phenolic Content (TPC) and Antioxidant Capacity of Rose Hip Flesh from Maturity

**Figure 3:**
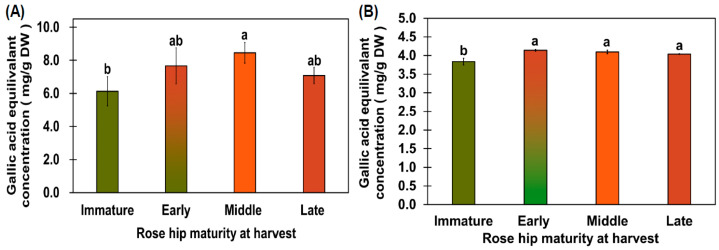
TPC (**A**) and DPPH capacity (**B**). TPC (**A**) and DPPH antioxidant capacity (**B**) were measured as gallic acid equivalents. The TPC of the 70% rose hip ethanol extract was measured at 715 nm using Folin–Ciocâlteu phenol reagent, whereas antioxidant activity was measured using 70% rose hip ethanol extract at 515 nm. Different letters above the error bars indicate significant differences according to Tukey’s HSD test (*p* < 0.05).

The immature flesh had a TPC of 6.13 ± 0.88 mg/g gallic acid equivalent (GAE) DW, and the early-, mid-, and late-ripeness flesh samples had TPCs of 7.67 ± 1.09, 8.45 ± 0.62, and 7.08 ± 0.49 mg/g GAE DW, respectively (Figure 3A). The early-, mid-, and late-ripeness rose hips did not show significantly different TPCs according to Tukey’s test (Figure 3A). Dolek et al. [24] reported that TPC and antioxidant activity were highly affected by a delayed harvest. In our study, TPC and antioxidant activity were not significantly different, except in immature rose hips. Koczka et al. [25] reported that the TPC of *R. spinosissima* ethanol extract was higher than that of *R. rugosa* ethanol extract. However, the TPC of the mid-ripeness rose hip was similar to that of the *R. spinosissima* ethanol extract. Based on previous studies and this study, early-, mid-, and late-ripeness rose hips have a high total phenol content. The flesh tissue from the immature rose hip fruit showed 3.84 ± 0.09 mg/g GAE DW of 2,2-Diphenyl-1-Picrylhydrazyl (DPPH) antioxidant capacity, whereas that from the early-ripeness stage showed 4.14 ± 0.03 mg/g GAE DW of DPPH antioxidant capacity, which was significantly higher than that of the immature stage (Figure 3B). The flesh samples from the early-, mid-, and late-ripeness rose hip stages did not show significantly different DPPH antioxidant capacities according to Tukey’s test (Figure 3B).

### 2.3. Elastase Inhibitory Activity

**Figure 4:**
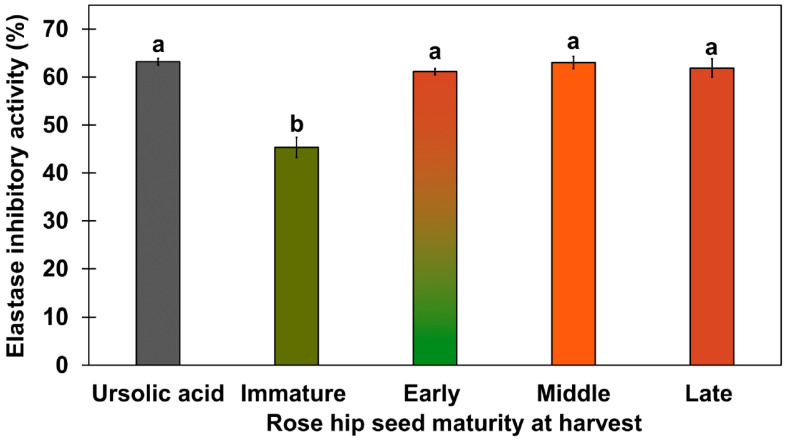
Elastase inhibitory activity of rose hip seeds from various ripening stages using 95% ethanol extract. Concentrations of freeze-dried rose hip seed samples were 400 µg/mL. The concentration of ursolic acid used for positive control was 50 µg/mL. Different letters above the error bars indicate significant differences according to Tukey’s HSD test (*p* < 0.05).

In a previous study, the elastase inhibitory activity of white rose petals and *Rosa centifolia* L. flower extract were evaluated [26,27]. Because previous studies focused on elastase inhibitory activity in flowers, our study focused on conducting an elastase inhibitory assay in rose hip flesh and seeds. In previous studies, elastase inhibitory activity has not been tested for rose hip flesh tissue alone. Thus, we tested the elastase inhibitory activity of rose hip flesh tissue; none of the samples showed any significant activity (Supplement Figure S1). *Rosa rugosa* seed extracts markedly inhibited elastase activity, although the 95% ethanol extracts of *R. rugosa* seeds showed higher elastase inhibitory activity than the 70% ethanol extracts of R. rugosa seeds. Therefore, active ingredients for elastase inhibitory activity may be non-polar compounds that are extractable by 95% ethanol (Figure 4; Supplement Figure S2). The 95% ethanol extracts of *R. rugosa* from the early-, mid-, and late-ripeness stages showed elastase inhibitory activity similar to that of 100 µg/mL ursolic acid. To our knowledge, this is the first study to show that *R. rugosa* seed extracts significantly inhibit elastase activity. To identify the active compounds in the elastase inhibitory activity in seeds, compounds in the non-polar fraction were identified using the National Institute of Standards and Technology (NIST) library (https://www.nist.gov/, accessed on 28 February 2020) [28] or ursolic acid standard (Sigma Aldrich, Saint Louis, MO, USA). A previous study reported that the ethanol extract of 500 µg/mL white rose petals has been shown to have an elastase inhibitory activity of less than 50% [26]. In our study, elastase inhibitory activity in 400 µg/mL rose hip seeds from early-, mid-, and late-ripeness stages had elastase inhibitory activity > 50%. Thring et al. [27] reported that a 90% ethanol extract of rose flowers had 24.15% elastase inhibitory activity. The elastase inhibitory activity of the 95% ethanol extract of mid-ripeness rose hip seeds in our study was two to three times higher than that of the ethanolic extract from white rose petals and flowers in a previous study [27]. Ying et al. [29] reported that 4–6 μM ursolic acid competitively inhibited human leukocyte elastase enzyme. Thus, we used ursolic acid as a positive control in the assay.

### 2.4. Water-Soluble Metabolite Profiling for Pathway Analysis

**Figure 5:**
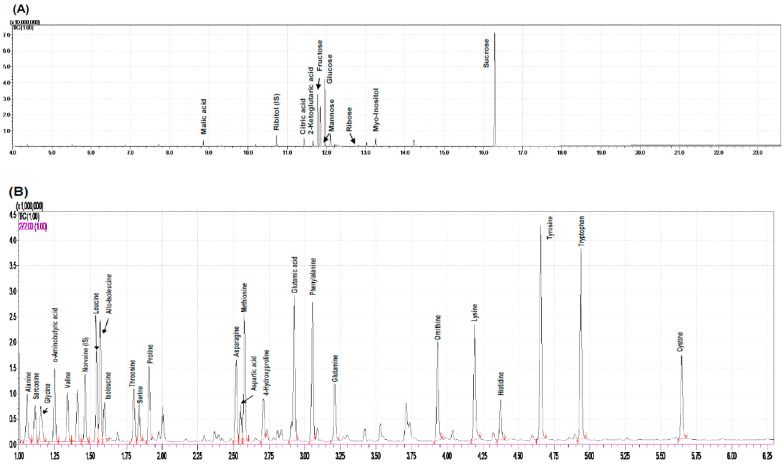

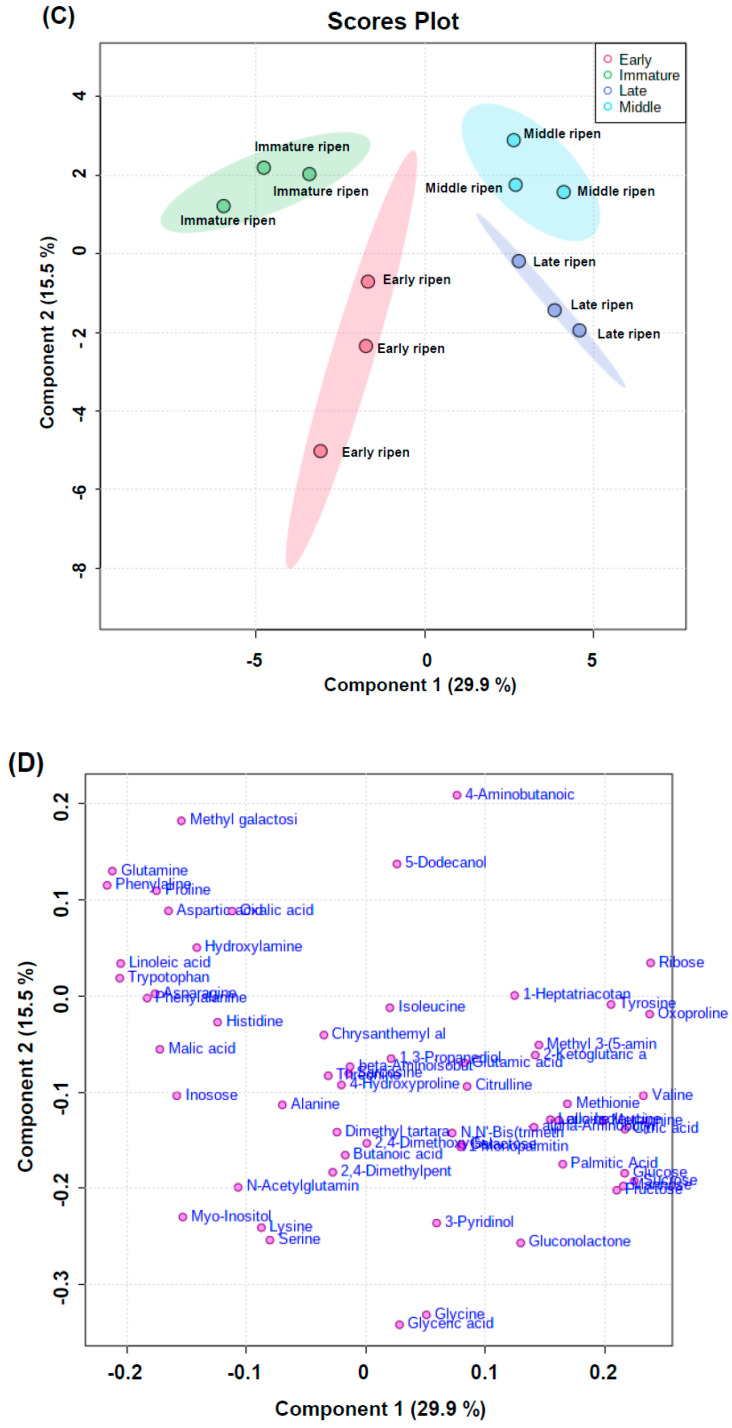
Chromatograms of water-soluble compounds (**A**) and amino acids from rose hip flesh (**B**) by GC-MS analysis. Partial least squares-discriminant analysis (PLS-DA) score plot (**C**) and loading plot (**D**) derived from GC-MS data of various ripening stages.

Based chromatograms of water-soluble compounds and amino acids from rose hip flesh by GC-MS analysis were used (Figure 5A,B). Water-soluble compounds and amino acids from rose hip flesh were quantified based on chromate grams.

**Figure 6:**
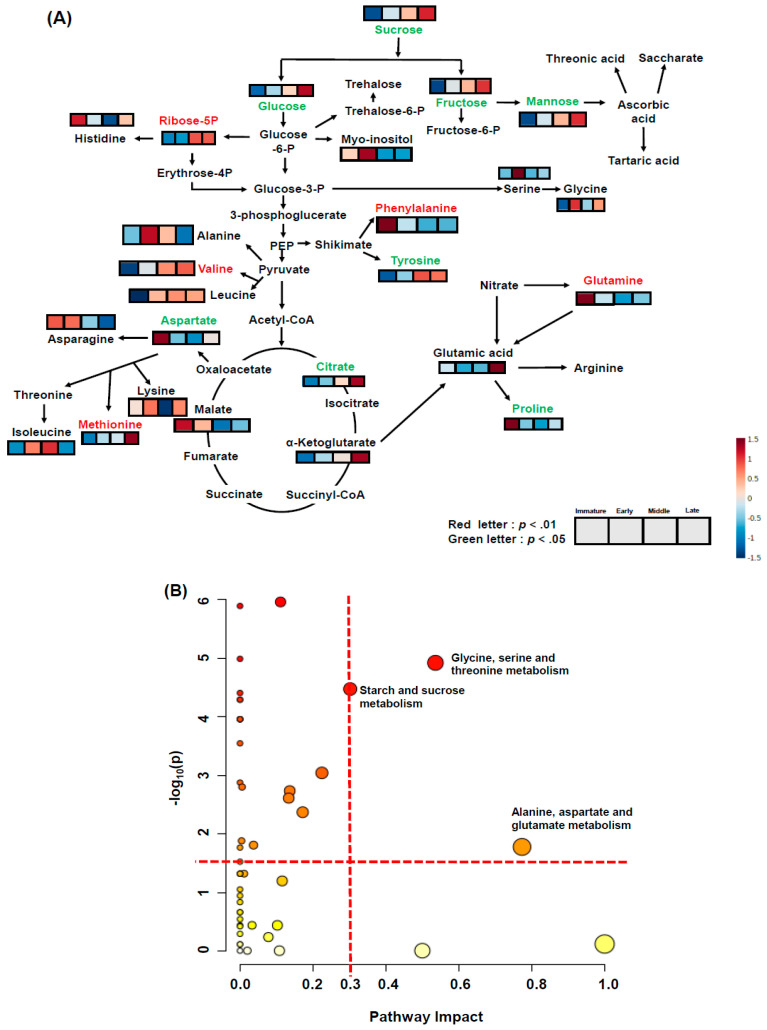
Global metabolomic profile of rose hip flesh at different maturity stages at harvest using gas chromatograph-mass spectrometry (**A**) and KEGG metabolism pathway analysis (**B**) of various ripening stages of rose hip flesh tissue. Significantly changed metabolites in the global metabolomic profile expressed in red (*p* < 0.01) or green letters (*p* < 0.05) above the heat map box according to Tukey’s HSD at *p* < 0.01 and 0.05, respectively. Significantly changed metabolic pathways were selected based on log10(*p*) value > 1.5 and impact value > 0.3 among different maturities at harvest.

Figure 5C,D present clustering patterns among various rose hip flesh maturity samples and their water-soluble metabolites at harvest using partial least-squares discriminant analysis (PLS-DA) score plots and loading plots, respectively. PLS-DA components 1 and 2 explained 29.9% and 15.5% of the total variance of the rose hip flesh primary metabolites, respectively (Figure 5C). The PLS-DA loading plot showed that the variables were correlated with Principal Component 1 and Principal Component 2 (PC1 and PC2) (Figure 5D). Figure 6A presents the relevant metabolites used as inputs for the heat map of the rose hip flesh. The metabolic pathways were based on the Kyoto Encyclopedia of Genes and Genomes (KEGG) analysis. The amino acid content and water-soluble compounds were expressed in the glycolysis, tricarboxylic acid (TCA) cycle, and their related pathway. The metabolites of sucrose, fructose, glucose, mannose, tyrosine, aspartate, citrate, and proline were significantly different depending on the maturity stage at harvest (*p* < 0.05, Figure 6A). Sugar concentrations gradually increased during ripening. The fructose and glucose contents of *R. dumalis* and *R. rubiginosa* fruit reportedly increase with maturity at harvest and decrease after excessive maturity at harvest [22]. In our study, fructose and glucose levels in rose hip flesh increased during ripening. Similarly, the metabolites for ribose-5P, phenylalanine, glutamine, and methionine were significantly different among the maturity stages at harvest (*p* < 0.01). Metabolites of histidine, myo-inositol, serine, glycine, glutamic acid, alanine, leucine, asparagine, lysine, and leucine were not significantly different at harvest. The significantly different water-soluble metabolites were expressed as ribitol equivalent concentrations (internal standard, Supplementary Figure S3). Pathway analysis was performed using water-soluble metabolites from mature rose hip flesh at harvest (Figure 6B). Three metabolic pathways (glycine, serine, and threonine metabolism; starch and sucrose metabolism; alanine, aspartate, and glutamate metabolism) during maturity at harvest significantly changed the metabolisms (−log10(p) value > 1.5 and impact value > 0.3). A previous study revealed that changes in starch and sucrose metabolism were caused by sugar changes during ripening [22,30]. Lee et al. [31] reported that amino acids such as aspartate, phenylalanine, lysine, serine, and proline during early maturation affected fruit development through enhanced respiration. Thus, aspartate, serine, and lysine are speculated to be involved in glycine, serine, and threonine metabolism; alanine, aspartate, and glutamate metabolism may be used for fruit development.

**Figure 7:**
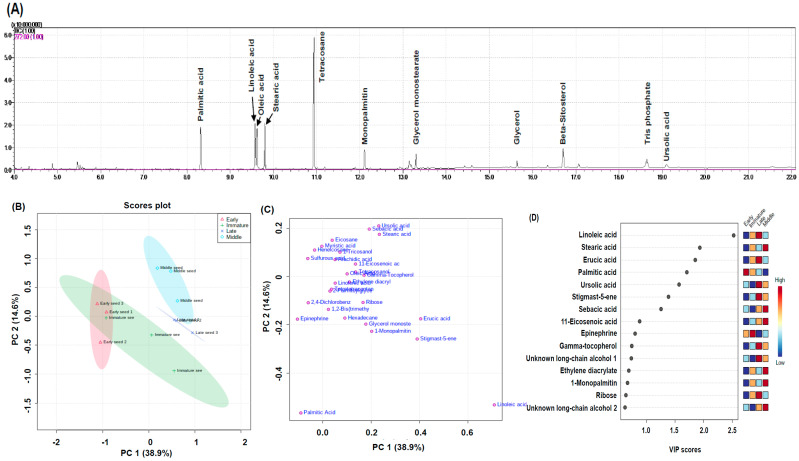
Chromatograms of lipid-soluble compound (**A**) from rose hip seeds by GC-MS analysis. Partial least squares-discriminant analysis (PLS-DA) score plot (**B**), loading plot (**C**), and variable importance in projection (VIP) scores (**D**).

Chromatograms of lipid-soluble compounds from rose hip seeds by GC-MS analysis were used (Figure 7A). Quantification of lipid-soluble rose hip seeds was based on chromate grams.

Figure 7B,C present clustering patterns among various rose hip seed maturity samples and their lipid-soluble metabolites at harvest using partial least-squares discriminant PC1 and PC2, which explained 38.9% and 14.6% of the total variance of rose hip seed primary metabolites, respectively (Figure 7B). The PLS-DA loading plot showed that the variables were correlated with PC1 and PC2 (Figure 7C). Seven metabolites of linoleic acid, stearic acid, erucic acid, palmitic acid, ursolic acid, stigmast-5-ene, and sebacic acid had the highest variable important in projection (VIP) score (>1) (Figure 7D).

**Figure 8:**
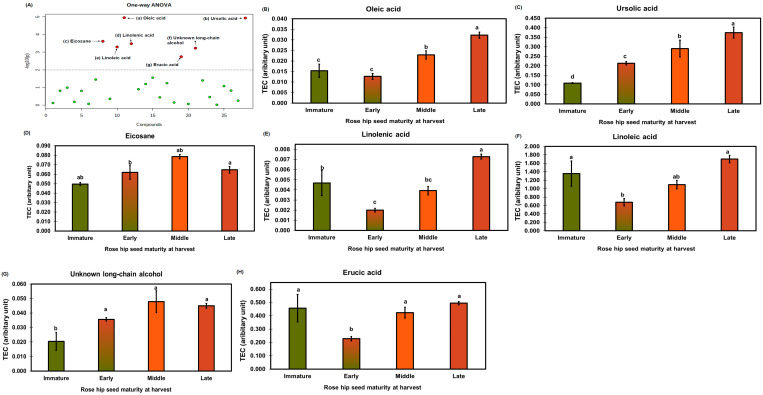
Red dots in the one-way ANOVA (**A**) indicate significantly different lipid-soluble metabolites among different rose hip maturities (*p* < 0.01). The specific concentrations of the lipid-soluble metabolites from rose hip seed (**B**–**H**) are expressed as a bar graph. TEC indicates tetracosane equivalent concentration based on an internal standard (tetracosane). The bars and error bars represent the mean ± standard deviation of three biological replications. Letters above bars represent significant differences among different rose hip maturities Tukey’s HSD test (*p* < 0.05).

The metabolites of rose hip seeds were analyzed using a one-way ANOVA (Figure 8A) followed by Tukey’s HSD test (*p* < 0.01). Relative concentrations of significantly different lipid-soluble metabolites were expressed as tetracosane equivalent concentrations in Figure 8B–H. Oleic acid showed the greatest significant difference in ripening compared to the other compounds (*p* < 0.01). Ursolic, linolenic, and linoleic acid levels were also highly significantly different throughout the ripening process (*p* < 0.01). Previous studies report that ursolic acid and C18 unsaturated fatty acids (e.g., linoleic and linolenic acids) are active compounds known to have elastase inhibitory activity [32,33]. The elastase inhibitory activity of eicosane and erucic acid has not previously been reported. The ursolic acid concentration of late rose hip seeds was the highest (689.97 ± 21.32 μg/g DW) (Supplementary Figure S4). Ursolic acid was not detected in rose hip flesh tissue, explaining why seeds have a higher elastase inhibitory activity than flesh (Supplemental Figure S2). Wenzing et al. [34] reported that ursolic acid and other triterpenes were detected in the powder of rose hip whole fruit (*R. canina* L.). However, which tissues contain the highest ursolic acid concentration has not been determined. Our study and previous studies found ursolic acid in the seeds of rosehip fruit. In addition, the different ursolic acid concentrations in rose hip seeds at various ripening stages partially explain the different elastase inhibitory activities in the immature stage and the other three ripeness stages (Figure 4). The relative concentrations of tetracosane may reveal a synergetic effect between ursolic acid and linoleic acids on elastase inhibitory activity.

## 3. Materials and Methods

### 3.1. Plant Materials and Preparation

Rose hips (*R. rugosa*) from the Chonnam National University Experiment Field (Gwangju, Republic of Korea) were used in this study. Rose hips were classified into immature, early-, mid-, and late-ripeness stages based on the red color of the fruit skin and their calyx (number of immature rose hip samples = 14; number of early-, mid-, and late-stage rose hip samples = 16). The calyx from the rose hip was removed and separated into flesh and seeds. The hips were then freeze-dried using an MCFD freeze-dryer (IlShinBioBase, Dongducheon, Republic of Korea). The dried samples were ground using an MM301 mixer mill (Retsch, Haan, Germany) for 1 min at 30 Hz and stored at −20 °C until sample extraction.

### 3.2. Fruit and Seed Color, Diameter, Weight, and Length Measurements

The red, green, and blue (RGB) values of all *R. rugosa* fruit flesh surfaces and calyxes were measured using an OI Color Picker (https://shaeod.tistory.com/565, accessed on 11 February 2021) from photographs taken using an SAL 1650 camera (Sony, Tokyo, Japan). The RGB values were individually measured for the fruit surface and the calyx (*n* = 10–14). After measurement, the R, G, and B were divided by 255 to adjust the value from 0 to 1, which is equivalent to Hunter’s LAB (lightness, Red/Green value, Blue/Yellow value) values using the tool (http://colormine.org/convert/rgb-to-hunterlab, accessed on 3 May 2021). Subsequently, the hue angle was determined by converting the RGB values based on the following equations (https://stackoverflow.com/questions/23090019/fastest-formula-to-get-hue-from-rgb, accessed on 3 May 2021):

R′ = R/255;(1)

G′ = G/255;(2)

B′ = B/255(3)

If the red value was the maximum among the RGB values, then

hue = (G − B)/(max − min).(4)

If the green value was the maximum among the RGB values, then

hue = 2.0 + (B − R)/(max − min).(5)

If the blue value was the maximum among the RGB values, then

hue = 4.0 + (R − G)/(max − min).(6)

The diameter and length were measured using a pair of digimatic calipers (Mitutoyo, Kanagawa, Japan). Weight was measured using an electronic scale IB-6100S (Innotem, Seoul, Korea). The parameters for the immature stage were measured in 10 rose hips; the parameters for early-, mid-, and late-ripeness stages were measured in 14 rose hips.

### 3.3. TPC Determination

Freeze-dried samples (50 mg) were extracted in 1 mL of 70% ethanol at 100 °C for 10 min using a Maxtable^TM^ H20 heating block (Daihan Scientific, Wonju, Republic of Korea). During heating, the samples were vortexed every 5 min. After heating, the tubes were cooled on ice for 15 min. The tubes were vortexed and centrifuged (Smart 15 Plus, Hanil, Gimpo, Republic of Korea) at 13,475× *g* for 2 min at room temperature (24 °C), and the supernatants were collected in new tubes. Samples from the immature stage were extracted from 14 rose hips; samples from the early-, mid-, and late-ripeness stages were extracted from 16 rose hips. TPC was analyzed following the method described by Ku et al. [35], with minor modifications. TPC was used to measure the antioxidant capacity of the rose hip flesh samples. Reaction mixtures containing 10 µL of the test samples or positive controls (gallic acid, Sigma Aldrich, Saint Louis, MO, USA) and 100 µL of 0.2 M Folin–Ciocâlteu phenol reagent (Sigma Aldrich, Saint Louis, MO, USA) in ethanol were incubated at room temperature for 3 min in 96-well plates. After 3 min, 100 µL of 7.5% Na_2_CO_3_ was added, followed by incubation at room temperature for 30 min. The absorbance was measured at 715 nm using a Spectra Max ABS Plus plate reader (Molecular Devices, San Jose, CA, USA). The results were expressed as gallic acid equivalent concentrations (GAE) (mg/g dry weight (DW)). The samples were assayed in triplicate.

### 3.4. The 2,2-Diphenyl-1-picrylhydrazyl (DPPH) Antioxidant Capacity

Sample extract used in the DPPH assay was obtained by the same method as the above-mentioned TPC assay. DPPH antioxidant capacity was analyzed following the method described by Ku et al. [35], with minor modifications. The DPPH assay was used to measure the antioxidant capacity of the rose hip flesh samples. Reaction mixtures containing 10 µL of the test samples or positive controls (gallic acid, Sigma Aldrich, Saint Louis, MO, USA) and 190 µL of 400 µM DPPH (optical density adjusted to 1.00, Sigma Aldrich) in ethanol were incubated at room temperature for 30 min in 96-well plates. The absorbance was measured at 515 nm using a Spectra Max ABS Plus plate reader (Molecular Devices, San Jose, CA, USA). The results are expressed as the GAE concentration (mg/g DW). The samples were assayed in triplicate.

### 3.5. Elastase Inhibitory Activity

Melan-a melanocytes are a highly pigmented, immortalized, standard murine melanocyte cell line derived from C57BL/6 mice. The melan-a cells used in the present study were obtained from Dr. Dorothy Bennett (St. George’s Hospital, London, UK). The cells were cultured at 37 °C in an atmosphere of 95% air and 10% CO_2_ in RPMI-1640 medium (Gibco BRL, Thermo Fisher Scientific, Seoul, Korea) and supplemented to a final concentration with 10% heat-inactivated fetal bovine serum, 1% penicillin/streptomycin, and 200 nM 12-o-tetradecanoylphorbol-13-acetate. Cell viability was determined using a CCK-8 cell counting kit-8 (Dojindo Lab, Kumamoto, Japan). Monolayers of confluent melanocytes were harvested using a mixture of 0.05% trypsin and 0.53 mM EDTA (Gibco BRL, Thermo Fisher Scientific, Seoul, Korea). Elastase inhibitory activity was treated with various solvents of Haematococcus extracts with 4.4 mM N-succinyl-(Ala)3-p-nitroanilide (S4760, Sigma Aldrich, Saint Louis, MO, USA) in 100 mM Tris-HCl (pH 8.0) on a 96-well plate (SPL, Pocheon-si, Gyeonggi-do, Korea); 1 unit/mL of elastase (E0258, from porcine pancreas, Sigma Aldrich, Saint Louis, MO, USA) was reacted for 20 min at 25 °C with 100 mM Tris-HCl (PH 8.0). The absorbance was measured at 405 nm using a microplate reader (3550 Microplate Reader, Bio-Rad, Hercules, CA, USA).

### 3.6. Water- and Lipid-Soluble Primary Metabolite Profiling Using Gas Chromatography–Mass Spectrometry (GC-MS)

Water- and lipid-soluble primary metabolites were analyzed according to the methods described by Lisec et al. [36]. A total of 50 mg of rose hip flesh and seed was weighed into 2 mL tubes. The samples were shaken at 60× *g* for 10 min with 1.4 mL methanol from the freezer and 50 µL of 10 mg/mL ribitol as an internal standard. Subsequently, the sample extract was centrifuged at 10,000× *g* for 3 min. A total of 700 µL of the supernatant from the centrifuged sample was transferred to 2 mL microcentrifuge tubes. The supernatant was vortexed for 10 s with 375 µL of ChCl_3_ and 700 µL of H_2_O from the freezer. The supernatant was centrifuged at 10,000× *g* for 3 min. A total of 50 µL of water-soluble compounds was transferred to 1.5 mL tubes. The samples were placed in a SpeedVac vacuum concentrator (Vision, Bucheon, Gyeonggi-do, Korea) at 30 °C for 1 d. Then, 100 μL of N,O-bis (trimethylsilyl) trifluoroacetamide (BSTFA) with 1% trifluoroacetamide (TMCS) was added, and the mixture was incubated at 60 °C for 90 min. The SpeedVac samples were incubated at 800× *g* for 90 min at 37 °C with 50 μL of freshly prepared methoxyamide in pyridine. The sample was incubated at 800× *g* for 20 min at 50 °C with 80 μL of N-Methyl-N-(trimethylsilyl)trifluoroacetamide (MSTFA).

For water-soluble metabolite analysis after an initial temperature of 80 °C for 2 min, the oven temperature was increased by 15 °C per min to 330 °C and held for 5 min. For lipid-soluble metabolite analysis, the temperature was increased by 12 °C per min to 320 °C and held for 7 min. The injector and detector temperatures were set at 205 and 250 °C, respectively. An aliquot (1 μL) of the sample was injected at a split ratio of 70:1. The carrier gas (helium) was maintained at a constant flow rate of 1.2 mL/min. The mass spectrometer was operated in positive electron impact mode at an ionization energy of 70.0 eV and a scan range of 40–500 *m*/*z* [37]. Metabolite identification was based on the National Institute of Standards and Technology (NIST) library [28]. The NIST library was used for water- and lipid-soluble compound identification of rose hip flesh and seed (Supplement Tables S1 and S2; Supplement Figure S1A,B). Significantly different metabolites from the lipid-soluble phase of rose hip seed were quantified using the relative response factor between the internal standard (tetracosane). Ursolic acid was used for compound identification standard compounds.

### 3.7. Amino Acid Quantification

To quantify the free amino acid content in the samples, an EZ:faast free amino acid of rose hip flesh for GC-MS kit (Phenomenex, Torrance, CA, USA) was used to extract and measure the amino acid concentration. A sample (50 mg) was extracted with 1 mL of 0.1 M HCl using an MM301 mixer mill (Retsch, Haan, North Rhine-Westphalia, Germany). After extraction, the samples were centrifuged at 9350× *g* for 10 min. Amino acid purification and derivatization were performed according to the manufacturer’s instructions. Amino acid analysis was performed using a gas chromatograph (GC-MS-QP2020 NX, Shimadzu, Kyoto, Japan) coupled to an electron impact and an autosampler (AOC-20S Plus, Shimadzu, Kyoto, Japan). A capillary column (ZB-AAA EZ-AAA amino acid Analysis GC, Phenomenex, Torrance, CA, USA; 10 m × 0.25 mm) was used. The injection ratio was 1:10, and the injection temperature was 250 °C. The injection volume was 2.0 µL. The carrier gas was helium, and the flow rate was 1.1 mL/min. The column temperature was set at 110 °C, and the temperature was increased by 30 °C per min to 320 °C. The MS temperature was set at 240 °C, and the scan range was set to 45–450 *m*/*z*. Standard compound mixtures and the National Institute of Standards and Technology (NIST) library [28] were used for amino acid compound identification (Supplement Figure S1C). Quantification amino acid was used for the calibration curve by injected standard compound mixtures 1 and 2 at a concentration of 0.25, 0.5, and 1 mM.

### 3.8. Data Processing and Statistical Analysis

All analyses were performed in triplicate (number of immature rose hip samples = 14; number of early-, mid-, and late-stage rose hip samples = 16). GC-MS data were processed with GC-MS solution software (Shimadzu, Kyoto, Nakaguo-ku, Japan) to integrate peaks. Peak areas were normalized to internal standard area (ribitol for water soluble; tetracosane for lipid soluble). Mid-ripeness rose hip flesh and seeds representative of all the rose hip flesh and seed samples were extracted and used for the optimization and validation tests. A large set of authentic standards were used for quality control by adding the first and last sequence of the metabolomic analysis to check the retention time stability and peaks intensity during GC-MS analysis. The datasets obtained by GC-MS comprised 24 amino acids from rose hip flesh, 36 water-soluble phase primary metabolites from rose hip flesh, and 28 lipid-soluble phase primary metabolites from rose hip seeds. Statistical analyses were conducted to compare group means, with significant differences (*p* < 0.05) between group means determined using Tukey’s honest significant difference (HSD) test in Prism 5 (GraphPad, Northside, San Diego, CA, USA). Partial least-squares discriminant analysis (PLS-DA), variable importance in projection (VIP) score, one-way analysis of variance (ANOVA), and heat maps were performed on the data after auto (rose hip flesh) and Pareto scaling (rose hip seed) using MetaboAnalyst 4.0 [38]. For rose hip flesh, auto scaling was used to evaluate pathway changes with equal weight for each variable; Pareto scaling was used for rose hip seeds to elucidate active compounds for elastase inhibitory activity by maintaining the original data structure.

## 4. Conclusions

In the present study, we analyzed the changes in metabolites and elastase inhibition resulting from different rose hip maturity stages at harvest. The primary metabolite levels of rose hip flesh resulted in significantly different sugar metabolism based on the maturity stage at harvest. The early-, mid-, and late-ripeness rose hip seeds showed high elastase inhibitory activity. Our study found that rose hip seeds have elastase inhibitory activity. Fatty acids and ursolic acid explained why the 95% ethanol extract had a higher elastase inhibitory activity than the 70% ethanol extract. Multiple regression and the literature show that fatty acids and ursolic acid may be the active compounds in elastase inhibitory activity. These findings suggest that rose hip seeds at the mid- and late-ripeness stages are ideal for use in the cosmetic industry to reduce wrinkles (wrinkle care products) by way of elastase inhibitory activity.

## Data Availability

All data generated or analyzed during this study are included in this published article.

## Supplementary Materials

The following are available online at https://www.mdpi.com/article/10.3390/plants10071283/s1.
